# European Inter-Laboratory Proficiency Test for Dourine Antibody Detection Using the Complement Fixation Test

**DOI:** 10.3390/vetsci10100592

**Published:** 2023-09-26

**Authors:** Laurent Hébert, Delphine Froger, Anthony Madeline, Fanny Lecouturier, Charlène Lemans, Stephan Zientara

**Affiliations:** 1 Unité Physiopathologie et Epidémiologie des Maladies Equines (PhEED), Laboratoire de Santé Animale, Site de Normandie, Agence Nationale de Sécurité Sanitaire de l’Alimentation, de l’Environnement et du Travail (ANSES), RD675, 14430 Goustranville, France; delphine.froger@anses.fr (D.F.); anthony.madeline@anses.fr (A.M.); fanny.lecouturier@anses.fr (F.L.); charlene_lemans@live.fr (C.L.); 2 UMR 1161 Virologie, Laboratoire de Santé Animale, Site de Maisons-Alfort, ANSES, Institut National de Recherche Pour l’Agriculture, l’Alimentation et l’Environnement (INRAE), Ecole Nationale Vétérinaire d’Alfort (ENVA), 94700 Maisons-Alfort, France; stephan.zientara@anses.fr

**Keywords:** dourine, complement fixation test, horse, inter-laboratory proficiency test

## Abstract

**Simple Summary:**

Dourine is a parasitic disease affecting equids, caused by *Trypanosoma equiperdum*. Thanks to the implementation of stamping out policies during the 20th century, dourine is now found only in few parts of the world. To prevent the spread of dourine, it is necessary to check that equids are not infected during international movements. In the context of the European Reference Laboratory for equine diseases mandate, our responsibility is to evaluate the capacity of the network of National Reference Laboratories to perform the prescribed test for dourine diagnosis: the complement fixation test. For this purpose, we organised inter-laboratory proficiency tests for dourine diagnosis; these consist of sending panels of samples of known status (containing or not containing anti-*T. equiperdum* antibodies) to determine whether the laboratories correctly identify the samples. The present article describes the results obtained by national reference laboratories during three sessions of dourine proficiency tests organised in 2015, 2018 and 2022, respectively. These sessions allow the identification of a reagent that should not be used for dourine serodiagnosis and help to improve the performance of the network. This article highlights the importance of inter-laboratory proficiency tests to improve the performance of laboratories and ensure the health safety of equids.

**Abstract:**

Dourine is a sexually transmitted parasitic disease affecting equids. Its causative agent is referred to as *Trypanosoma equiperdum* and the prescribed serodiagnosis method is the complement fixation test (CFT). In the context of our European Reference Laboratory mandate for equine diseases (excluding African horse sickness), we organised dourine CFT inter-laboratory proficiency tests (ILPTs) in 2015, 2018 and 2022 to evaluate the performance of the European Union network of National Reference Laboratories (NRLs) for dourine. ILPT panels were composed of horse sera with or without antibodies against *Trypanosoma* spp. originating from non-infected, immunised or experimentally infected horses. Twenty-two NRLs participated in at least one of the three sessions. In 2015, 2018 and 2022, the percentage of laboratories obtaining 100% of the expected results was 57, 90 and 80, respectively. These dourine CFT ILPTs showed the benefits of standardising the method’s detection limit and underlined the constant need to evaluate NRLs to improve the network’s performance. These results also argue in favour of the need for a representative bio-bank to improve the representativeness of ILPT samples and to allow the adoption of alternative serological methods for international surveillance of dourine.

## 1. Introduction

Dourine is a contagious parasitic disease affecting equids that is mainly transmitted from animal to animal during coitus. During the first phase of the disease, the parasite invades the animal’s tissues and blood, causing the appearance of clinical symptoms such as fever, local oedema, emaciation and anaemia [1]. After this initial invasion phase, the parasite may reach the animal’s central nervous system [2,3] and cause clinical signs of nervous system disorders (e.g., lack of coordination or paralysis) leading to the animal’s death. Dourine cases have to be reported to the World Organisation for Animal Health (WOAH, previously OIE) and the European Commission for European countries.

According to international regulations [1], the causative agent of dourine belongs to the *Trypanozoon* subgenus and the *Trypanosoma equiperdum* species. In recent years, analysis of the genomes of members of the *Trypanozoon* subgenus—which also includes the parasites responsible for nagana (*Trypanosoma brucei brucei*) and surra (*Trypanosoma brucei evansi*)—has shown that this division into three different subspecies is inadequate because these names gather genetically distinct (polyphyletic) parasites [4]. To date, no consensus has emerged on how to reconcile pathologies and phylogeny [5,6]; the biological context remains the most common determinant for the designation of newly isolated strains [7].

Currently, equine trypanosomosis in general and dourine in particular remain endemic in parts of Africa [8,9], Asia [10,11] and Latin America [12,13,14]. Apart from sporadic cases [15,16], many parts of the world are considered free of equine trypanosomosis, including Europe and North America [17]. At present, the differential diagnosis of dourine, surra and nagana in horses remains challenging [17,18] due to (i) the absence of pathognomonic clinical signs of dourine, surra and nagana, (ii) the incongruity between the disease classification used by WOAH and the numerous genetic groups within the *Trypanozoon* subgenus [17] and (iii) the fact that parasitaemias are usually below the detection limit of parasitological and molecular tests [19].

In this context, dourine surveillance during international trade is based on the WOAH’s recommendation of the complement fixation test (CFT) [1]. This technique is based on the use of raw *T. b. equiperdum* antigens, but given the genetic proximity between the different members of the *Trypanozoon* sub-genus, the CFT cannot be considered specific to dourine, as it will also reveal infections by other trypanosomoses such as *T. b. brucei* and *T. b. evansi* [17]. CFT performance can also be affected by the quality of the reagents used (complement, erythrocytes, antigens, etc.), which also require careful titration.

In Europe, dourine surveillance is coordinated by a network of National Reference Laboratories (NRLs). In 2008, the European Commission appointed the French agency for food, environmental and occupational health and safety (ANSES) as the European Reference Laboratory (EURL) for equine diseases (excluding African horse sickness) with the remit of harmonising and improving the diagnosis of equine diseases in Europe. After two dourine CFT ILPTs organised by ANSES in 2009 and 2012 [20], the present study was designed to evaluate the performance of European Union NRLs for dourine sero-diagnosis using the CFT [1]

## 2. Materials and Methods

### 2.1. Panel Description

Dourine CFT ILPT panels were composed of horse sera deliberately chosen to contain, or not to contain, antibodies against *Trypanosoma* spp. (Table 1). Panels were randomly coded and shipped at +2–8 °C to participants. Samples were not previously heat-inactivated, and needed to be stored at +2–8 °C to avoid repeated thawing/freezing cycles. The stability of each freeze-dried sample was confirmed by performing accelerated ageing condition tests showing that no variation in dourine CFT results was observed following 3 weeks of incubation at 30 °C. In addition, the panels’ stability was validated by analysing, for each ILPT session, one panel stored at 4 °C throughout the ILPT period and tested at the end of it.

### 2.2. Participating Laboratories

**Figure 1:**
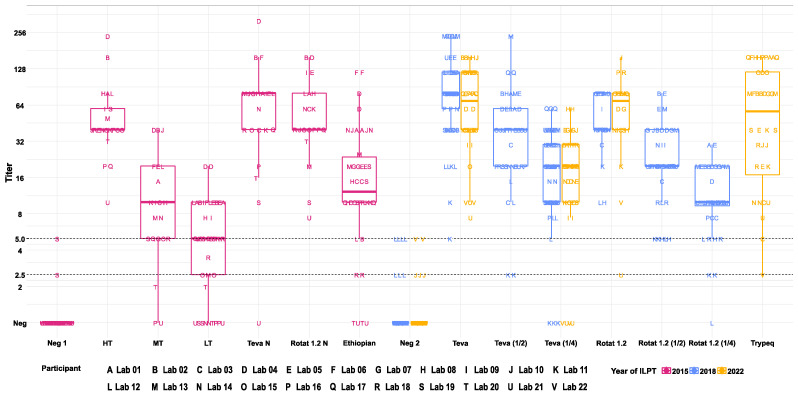
Box plot representing the distribution of titres obtained with the dourine CFT by NRLs during the 2015, 2018 and 2022 ILPTs according to the year and serum ID. Titres were calculated as described in the material and methods section. The threshold values for considering a serum doubtful was 2.5 < titre < 5 and the positivity threshold was >5; they are indicated by horizontal dashed lines.

Twenty-two European Union dourine NRLs participated in at least one of the three dourine CFT ILPT sessions organised in 2015, 2018 and 2022 (Figure 1, Table 2 and Table S1). During each dourine CFT ILPT session, a random code number was assigned to each participant to ensure a blind analysis of the results. For the purpose of this article, each dourine NRL was identified with a number from 1 to 22 in order to monitor laboratory performance over the three dourine CFT ILPT sessions (Table 2). In order to prevent any risk of collusion, ANSES organised these ILPTs but did not participate as a French NRL despite being an EURL for equine diseases.

### 2.3. 2015 Sera Panel

Panels consisted of 150-µL aliquots containing different serum samples (Table 1). These sera, kept frozen for long-term storage, were thawed and divided into different vials for panel preparation and shipped at 4–8 °C. Participants were given 2 weeks to send back their results.

### 2.4. 2018 and 2022 Sera Panel

The panels consisted of freeze-dried sera. Each test entity had to be stored at +2–8 °C before and after reconstitution. Each serum had to be reconstituted with 0.5 mL of distilled water, vigorously mixed and then incubated for 30 min at room temperature with occasional mixing until fully dissolved. Participants were given 4 weeks to send back their results. Before sending the samples to each participant, the homogeneity was validated following ISO 17043 requirements [21]: ten aliquots of each of the samples were analysed in duplicates to check whether the qualitative results were the same for each aliquot containing the same serum.

### 2.5. EURL CFT Protocol

The participants were asked to test the samples using the dourine CFT according to the diagnostic methods they routinely applied in their laboratories. The results had to be read as per chapter 3.6.3 of the WOAH Terrestrial Manual [1] based on the degree of haemolysis inhibition (HI) expressed as follows: 0, trace, 1+, 2+, 3+ or 4+ (corresponding to 0%, trace, 25%, 50%, 75% or 100% of unlysed red cells, respectively) at 1/5 dilution with qualitative results determined at the 1/5 dilution as follows:

- HI of 0 or trace: Negative serum (Neg),
- HI of 1+: Suspicious serum (Susp),
- HI of 2+, 3+, 4+: Positive serum (Pos),
- serum with anti-complementary activity: Inconclusive serum (IC).

Some laboratories provided qualitative results that did not comply with this reading grid; their results were analysed on the basis of the results they provided. All sera with a positive reaction at 1/5 dilution could be serially diluted (rate 2) and tested using the dourine CFT to define their positivity cut-off point. In order to facilitate the analysis of quantitative results, this positivity cut-off point was translated into titres according to the following formula:

Titre=Last dilution with % of HI ≥ 50% × % of HI of the dilution × 2

As an example, a result of 3+ obtained at dilution 1/10 corresponds to a titre of: 10 × 75% × 2 = 15. According to this formula, the threshold positivity described by the WOAH terrestrial manual is 5 (5 × 50% × 2 = 5). All the results reported by the dourine CFT ILPT participants are presented in Table S1.

### 2.6. Analytical Methods

Participants were asked to specify the method used, the antigen supplier, the antigen batch number and expiry date, the suppliers of positive and negative controls and whether antigens from each new batch were titrated. The participants’ results were compared with those obtained by the EURL during preliminary challenges (homogeneity and stability).

To evaluate the performance of the methods in different laboratories, their specificity, sensitivity, positive predicted value (PPV) and negative predictive value (NPV) were calculated according to the following formulas:

$$Sensitivity=\frac{\sum True\;(+)}{\sum True\;(+)+\sum False\;(-)}$$

$$Specificity=\frac{\sum True\;(-)}{\sum True\;(-)+\sum False\;(+)}$$

$$PPV=\frac{\sum True\;(+)}{\sum True\;(+)+\sum False\;(+)}$$

$$NPV=\frac{\sum True\;(-)}{\sum True\;(-)+\sum False\;(-)}$$

The box plot representing the distribution of dourine CFT titres was generated using R [22].

## 3. Results

### 3.1. Dourine CFT ILPT Organisation and Panel Samples

The panel sent to participants for the three dourine CFT ILPT sessions are described in Table 1. Due to the difficulty in accessing positive sera from naturally or experimentally infected horses, we were only able to include two sera from experimentally infected horses (Ethiopian and Trypeq) in the panels distributed during the different ILPT sessions. To complete the panels, we included sera from horses immunised with whole cell lysate (raw antigens) from different *Trypanozoon* strains. In order to obtain sera with varying titres, we diluted sera from immunised horses with negative sera, as described in Table 1.

Given that the dourine CFT is based on the use of raw antigens, cross-reactions are expected for any serum from an animal infected or immunised with parasites from the different *Trypanozoon* clades. As a consequence, positive results in the dourine CFT for sera from horses immunised with *T. b. evansi* type A antigens were considered an expected result.

During the 2015 session, sera were sent in native form (4 °C), whereas during the 2018 and 2022 sessions, freeze-dried serum panels were distributed. This allowed us to include four identical serum batches (Neg 2, Teva, Teva (1/4) and Rotat1.2) during ILPT 2018 and 2022 (Table 1). No problems with sample reconstitution were reported by participants.

### 3.2. Analysis of Results from the Dourine CFT ILPT Participants

Twenty-two European Union dourine NRLs participated in at least one of the three dourine CFT ILPT sessions: 21 in 2015, and 20 in 2018 and 2022 (Figure 1 and Table S1). Nineteen NRLS participated in all three dourine CFT ILPTs, one participated in two and two NRLs participated in one.

As the dourine CFT is considered a qualitative method in the WOAH Terrestrial Manual, only the positivity statuses of the panel sera were taken into account for the analysis of the results returned by the participants (Table 2 and Table S1). Of the 19 laboratories that participated in all three sessions, 8/19 (42%) obtained 100% of expected results in 3/3 sessions, 10/19 (53%) obtained 100% of expected results in 2/3 sessions and 1/19 (5%) obtained 100% of expected results in 1/3 sessions.

During the 2015 dourine CFT ILPT session, 12/21 (57%) laboratories obtained 100% of expected results while 9/21 (43%) obtained between two and three unexpected results. Out of these nine laboratories, one encountered a problem of specificity (Lab 19) and nine encountered a problem of sensitivity. The sensitivity problem was encountered in particular with the two sera having low expected titres in the panel: LT serum (9/9) and MT serum (5/9). It can also be noted that the three laboratories using E-1 antigens obtained the highest number of unexpected results.

During the 2018 dourine CFT ILPT session, 18/20 (90%) laboratories obtained 100% of expected results while 2/20 (10%) obtained eight unexpected results. Of these two laboratories, one encountered a problem of specificity (Lab 12) and two a problem of sensitivity (Lab11 and Lab12). It can be noted that labs 11 and 12 had the highest antigen dilutions of all the 2018 session participants (160 and 1400, respectively).

During the 2022 dourine CFT ILPT session, 16/20 (80%) laboratories obtained 100% expected results while 4/20 (20%) obtained between one and six unexpected results. Of these four laboratories, two encountered a problem of specificity (Labs 10 and 22) and three a problem of sensitivity (Labs 9, 21 and 22). There was no clear link found during this session between the unexpected results obtained and antigen batch and dilution.

### 3.3. Analysis of Quantitative Results per Serum

The sera used to constitute the panels are described in Table 1, and the results returned by the participants are presented in Figure 1 and Table S1. During the 2015 session, 20/21 (95%) laboratories identified the Neg 1 samples as negative and one laboratory identified both Neg 1 replicates as “suspicious”. All laboratories identified the HT and Rotat 1.2 N sera as positive. Only Lab21 identified the Teva N sample as negative. Twelve out of 21 (57%) laboratories identified both MT and LT sera as positive, these being the sera with the lowest expected titres. Serum Ethiopian (the only one in the panel from an infected animal) was identified as negative by two participants and suspicious by one participant.

During the 2018 and 2022 sessions, three laboratories did not identify Neg 2 samples as negative, corresponding to a total of 12 (0.6%) unexpected results out of 200 Neg 2 samples analysed. As expected, for the sera obtained from horses immunised with the antigen of *T. b. equiperdum* Teva or *T. b. evansi* Rotat 1.2, the mean of the titres obtained by participants decreased with the dilution of the sera (Table S1). For the sera obtained from horses immunised with the antigen of *T. b. equiperdum* Teva or *T. b. evansi* Rotat 1.2, false negative results were more frequently obtained with the 1/4 dilution (7/100 [7%] and 3/40 [7.5%], respectively) than with the pure serum (0/100 [0%] and 2/40 [5%], respectively) or the 1/2 diluted sera (1/20 [2.5%], 0/40 [0%], respectively). For the Trypeq serum from experimentally infected horses, all the laboratories correctly identified the two duplicates as positive, with the exception of laboratories 9 and 22, which reported one and two replicates as suspicious, respectively.

## 4. Discussion

Although absent in many parts of the world, equine trypanosomosis in general, and dourine in particular, remain a threat that requires maintaining health controls during international trade [19,23]. In the European Union, the dourine NRL network ensures surveillance and can thus warn of the emergence of this disease. Among the tasks assigned to the EURL for equine diseases (other than African Horse Sickness) is that of evaluating the performance (sensitivity and specificity) of this network [24]. Because of the intermittent presence of trypanosomes in the blood of infected animals, serological checks are still preferred to microscopic or molecular methods, and the CFT remains the prescribed method for dourine diagnosis in the context of international trade [1]. In 2014, the analysis of the performance of the NRL dourine network concluded that dourine sero-diagnosis may be improved by standardising the critical reagents, and by developing standard *T. equiperdum* sera which could be used to calibrate tests [20]. The present article therefore describes the results obtained by the dourine NRLs in the European Union during their participation in the dourine CFT ILPT sessions held in 2015, 2018 and 2022.

During the ILPT sessions, the specificity of the CFT immunoassays performed by the participants was assessed by including several replicates of negative sera in the panels. Out of a total of 61 participants over the three sessions, four (6.6%) identified negative sera as positive or doubtful. We can observe that these laboratories returned unexpected results for several replicates within the same session and that the titres expressed remained close to the positivity threshold (no greater than 5). Thus, the most likely hypothesis is that these laboratories were faced with a problem of the titration of the elements in the complex—red blood cells/complement/haemolytic serum—resulting in the incomplete lysis of red blood cells even when there were no specific antibodies. It is therefore recommended to ensure effective lysis of red blood cells in the lysis control well of each serum (a well without any antigen); it may also be advisable to add a control well (without serum) in which the complement is diluted to 1/2 or even 1/4 to ensure that the quantity of complement is sufficient to guarantee the effective lysis of the red blood cells.

Serological tests aim to determine whether or not an individual has antibodies specific to a given pathogen. The setting of a positivity threshold is thus a key element that influences the sensitivity, specificity, NPV and PPV of a given test [25]. During CFT reagent titration procedures, the positivity threshold is established on the basis of the percentage of complement fixation obtained with a low-titre serum. Given that Europe has been free of dourine for decades (apart from an outbreak in Italy in 2011 [15]), it is a real challenge to obtain serum from infected animals representative of a low level of reactivity in the dourine CFT. To overcome this, laboratories generally use sera from horses immunised with *Trypanosoma* sp. crude antigens, which give a low signal in the dourine CFT. However, this means that there is a risk of establishing an artificial positivity threshold unrelated to thresholds encountered in naturally infected animals. In addition, the use of serum from infected horses enables the haemato-biochemical alterations that may occur during infection to be taken into account. This is why the Ethiopian serum from an experimental infection of horses with *T. b. equiperdum* Ethiopian strain [8] was included in the 2015 dourine ILPT. This serum was not identified as positive by three laboratories with a lack of sensitivity (laboratories 18, 20 and 21), highlighting the need to standardise thresholds. During the 2022 session, a freeze-dried serum from a horse experimentally infected with *T. equiperdum* OVI (Trypeq sample) was included. Of the 20 participants, none identified Trypeq samples as negative. However, two participants indicated suspicious results: participant 9, who obtained one positive and one suspicious result for the Trypeq sample duplicates (this laboratory also obtained lower levels of positivity than the other participants for all the sera, suggesting an overall problem of sensitivity) and participant 22, whose results showed a major problem of sensitivity (43%) and specificity (33%). It can also be noted that the titres obtained for these sera have a wide distribution range (from 2.5 to 160), suggesting that efforts are still required to standardise reagents and/or experimental practices across all participants.

For the 2018 session, the ILPT test components were made up of freeze-dried samples produced in relatively large batches, which meant it was possible to compare the laboratories’ performance over and between the different sessions. During the preparation of samples, dilutions were carried out for the Teva and Rotat 1.2 sera in order to obtain pure, half-diluted and quarter-diluted sera. An analysis of the results obtained by participants showed a good proportionality between the titres reported and the sera dilutions, demonstrating the CFT’s ability to provide quantitative results proportional to the levels of antibodies in the sera (Table S1).

During the 2015 dourine CFT ILPT session, we observed that 9/21 (43%) of laboratories had sensitivity problems, which were revealed in particular with the LT serum. Following this, to enable laboratories to set up their positivity thresholds, we produced and distributed among the EU laboratory network two control sera (a low titre and a high titre) together with an antigen produced from *T. b. equiperdum* OVI parasites propagated in rats. Following the implementation of these measures, we observed that the number of laboratories encountering sensitivity problems during the 2018 and 2022 sessions remained low: 2/20 (10%) and 3/20 (15%), respectively.

During a CFT, the specificity of the diagnosis is based on the antigen. In these ILPT sessions, participants used antigens of six different origins, three from in-house production and three from commercial production. We observed that the laboratories that used non-commercial antigens obtained 100% of the expected results. This observation suggests that laboratories capable of producing dourine antigens have sufficient expertise to carry out effective dourine diagnostics. In parallel, the three laboratories that used the commercial E-1 antigen each obtained unsatisfactory results for 50 to 60% of the tested samples. Because of these results, we advised participants against using this provider’s antigen, so it was no longer used by the NRLs in either the 2018 or 2022 sessions. For the other two commercial antigens (A and F), we did not observe a link between supplier, batch and participant performance. Even though some users of these antigens may have encountered difficulties during certain sessions, the small number of users of a given batch makes it difficult to establish a correlation between the use of an antigen and laboratory performance. Besides, it is interesting to note that for the same antigen batch, large disparities in antigen concentration can be observed (e.g., F-1 was used from 1/20 to 1/1400 and F-2 from 16 to 256), yet the results obtained do not appear to be affected by the dilution used.

Recently, phylogenetic analyses have shown that the causative agents of dourine, surra and nagana are polyphyletic [26] and, as an example, that clades of parasites responsible for dourine may be genetically closer to infectious agents of surra than to other infectious agents of dourine [5,6]. These considerations, which call into question the links between phylogeny and pathologies, have led to the suggestion that dourine could correspond to a clinical syndrome induced by a specific immune response of equids following infection by a *Trypanozoon* [17]. The fact that the CFT antigens consist of *Trypanozoon* whole cell lysate implies that this technique cannot be clade-specific and therefore cannot distinguish between dourine, surra and nagana [17]. In the context of health controls prior to international movements of equids, the fact that the analysis officially known as the dourine CFT can also detect animals suffering from surra or nagana (which are also regulated diseases) is not in itself a problem, as it helps to guarantee the health status of these equids. This is why positive dourine CFT results for sera from horses immunised with *T. b. evansi* Rotat 1.2 antigens (Table 1) were considered an expected result at the ILPT sessions.

## 5. Conclusions

As part of our EURL activities, ILPTs are held to assess the performance of the EU reference laboratories using routine dourine CFTs. In a context where, depending on the national organisation and importance of horse movements, the number of tests carried out by NRLs can vary from a few to several hundred per year, this evaluation is necessary because an erroneous result can have dramatic economic and/or sanitary consequences. The three ILPTs sessions organised enabled corrective action to be taken, in particular by advising against the use of a given batch of antigen, and made possible an overall improvement in the network’s performance following the distribution of a standard serum and antigens by the EURL. In the future, improving laboratory performance will involve disseminating user-friendly standard operating procedures, organising dourine CFT ILPT sessions including high quality serum from infected animals and organising training sessions for laboratories with deficiencies or for new laboratories wishing to start performing dourine CFT. To date, the main obstacle to improving and standardising the diagnosis of dourine (and equine trypanosomoses) is the difficulty in accessing representative field samples from different geographical areas (and therefore different clades of *Trypanozoon*) and different stages of the disease. The development of field sample banks would make it possible to determine precise detection thresholds on the basis of tangible biological data from infected animals rather than immunised animals; this would, in turn, maximise sensitivity without excessively reducing specificity. By creating such a bio-bank, it would also be possible to provide full field validation, according to the principles and methods applied to the validation of diagnostic assays for infectious diseases recommended by WOAH, for promising alternatives such as ELISA tests [27,28], Luminex [29] or ICT [30]. More generally, this study highlights the importance for any laboratory carrying out or wishing to carry out CFTs, whether for dourine or any other disease, to participate in external performance evaluation processes such as ILPTs in order to guarantee the maintenance of the laboratory’s performance.

## Figures and Tables

**Table 1:** Description of the 2015, 2018 and 2022 dourine CFT ILPT panels.

						Nbr of Repetitions per Panel		
Sample Origin	Serum ID	Dilution	Form	Dourine CFT Expected Result	Dourine CFT Titres from EURL Tests	2015	2018	2022
Mix of negative horse sera	Neg 1	-	Native	Negative	0	2		
Horse immunisation with *T. b. equiperdum* OVI antigens	HT	-	Native	Positive	60	1		
Horse immunisation with *T. b. equiperdum* OVI antigens	MT	-	Native	Positive	8	1		
	LT	3/5 dilution of MT serum	Native	Positive	5	2		
Horse immunisation with *T. b. equiperdum* Teva antigens	Teva N	-	Native	Positive	80	1		
Horse immunisation with *T. b. evansi* Rotat 1.2 antigens	Rotat 1.2 N	-	Native	Positive	40	1		
Horse experimental infection with *T. b. equiperdum* Ethiopian strain	Ethiopian	-	Native	Positive	20	2		
Mix of negative horse sera	Neg 2	-	Freeze-dried	Negative	0		7	3
Horse immunisation with *T. b. equiperdum* Teva antigens	Teva	-	Freeze-dried	Positive	80		3	2
	Teva (1/2)	1/2 dilution of Teva serum	Freeze-dried	Positive	40		2	
	Teva (1/4)	1/4 dilution of Teva serum	Freeze-dried	Positive	20		3	2
Horse immunisation with *T. b. evansi* Rotat 1.2 antigens	Rotat 1.2	-	Freeze-dried	Positive	60		1	1
	Rotat 1.2 (1/2)	1/2 dilution of Rotat 1.2 serum	Freeze-dried	Positive	30		2	
	Rotat 1.2 (1/4)	1/4 dilution of Rotat 1.2 serum	Freeze-dried	Positive	15		2	
Horse experimental infection with *T. b. equiperdum* OVI strain	Trypeq	-	Freeze-dried	Positive	80			2
				Number of samples per ILPT:		10	20	10

CFT, complement fixation test; EURL, European Union Reference Laboratory; ID, identifier; ILPT, inter-laboratory proficiency test.

**Table 2:** Analysis of the qualitative results obtained and the antigen used by the dourine CFT ILPT participants over the three sessions.

	2015				2018				2022			
	# of Unexpected Results	Successfully Identified Samples (%)	Antigen ^b^	Antigen Dilution	# of Unexpected Results	Successfully Identified Samples (%)	Antigen Provider	Antigen Dilution	# of Unexpected Results	Successfully Identified Samples (%)	Antigen Provider	Antigen Dilution
Participant 1	0	100	A-1	60	0	100	F-1	20	0	100	F-2	16
Participant 2	0	100	A-2	200	0	100	A-11	100	0	100	F-3	30
Participant 3	0	100	A-3	100	0	100	F-1	50	0	100	F-2	40
Participant 4	0	100	B-1	40	0	100	B-1	30	0	100	B-3	10
Participant 5	0	100	A-4	64	0	100	A-4	64	0	100	F-3	48
Participant 6	0	100	C-1	80	0	100	C-2	70	0	100	C-3	20
Participant 7	0	100	A-1	100	0	100	A-12	128	0	100	A-15	200
Participant 8	0	100	D-1	100	0	100	D-2	180	0	100	D-3	100
Participant 9	0	100	A-5	100	0	100	F-1	50	1	90	A-16	100
Participant 10	0	100	B-2	32	0	100	B-2	32	3	70	F-2	64
Participant 11	0	100	A-6	128	7	65	A-13	160	0	100	F-3	10
Participant 12	0	100	A-3	50	8	60	F-1	1400	-	-	-	-
Participant 13	2	80	A-7	50	0	100	F-1	30	0	100	F-2	30
Participant 14	2	80	A-3	10	0	100	F-1	40	0	100	F-2	256
Participant 15	2	80	A-8	100	0	100	F-1	30	0	100	F-2	30
Participant 16	3	70	A-3	15	0	100	F-1	60	0	100	F-2	75
Participant 17	3	70	A-9	40	0	100	A-14	50	0	100	F-2	20
Participant 18	3	70	A-10	200	0	100	F-1	30	0	100	F-3	30
Participant 19	5	50	E-1	128	0	100	F-1	30	0	100	A-17	20
Participant 20	5	50	E-1	16	-	-	-	-	-	-	-	-
Participant 21	6	40	E-1	32	0	100	A-12	100	3	70	A-18	9
Participant 22	- ^a^	-	-	-	-	-	-	-	6	40	F-3	10
% of laboratories obtaining 100% successful results		**57%**				**90%**				**80%**		

^a^ The laboratory did not participate in the ILPT session. ^b^ For confidentiality reasons, the names of the antigens are coded. Each letter refers to a supplier and each number refers to a production batch. The B, C and D antigens are produced in-house and the A, E and F antigens are commercially available. The shades of red are representative of the number of unexpected results obtained by the participants.

## Data Availability

The data presented in this study are available in Supplementary Table S1.

## Supplementary Materials

The following supporting information can be downloaded at: https://www.mdpi.com/article/10.3390/vetsci10100592/s1, Table S1: Results of the Dourine CFT ILPT per year and per participant.
